# Supplementary material for: Early experience affects foraging behavior of wild fruit bats more than their original behavioral predispositions
Source: eLife. 2025 Nov 11;14:RP103220. doi: 10.7554/eLife.103220 (PMC12604856; doi:10.7554/eLife.103220)
Supplement: Supplementary file 1. [file elife-103220-supp1.docx]

**Supplementary File 1.** Mixed GLM results for baseline trials of two environmental conditions

response ~ 1 + Environmental_condition + Trial_number + (1| Bat_ID)'

| Response | AIC | BIC | LogLikelihood | Deviance |  |  |  |  |
| --- | --- | --- | --- | --- | --- | --- | --- | --- |
| Boldness (N=40) | -74.127 | -62.28 | 42.064 | -84.127 |  |  |  |  |
|  | Fixed effects coefficients (95% CIs) | | | | | | | |
|  | Name | Estimate | SE | tStat | DF | P value | Lower | Upper |
|  | Intercept | 0.340 | 0.047 | 7.244 | 76 | 3.031e-10 | 0.246 | 0.434 |
|  | Environmental condition | -0.022 | 0.044 | -0.497 | 76 | 0.620 | -0.110 | 0.066 |
|  | Trial number (1 or 2) | 0.133 | 0.023 | 5.776 | 76 | 1.581e-07 | 0.087 | 0.178 |
| Response | AIC | BIC | LogLikelihood | Deviance |  |  |  |  |
| Boldness (N=19) | -30.614 | -22.559 | 20.307 | -40.614 |  |  |  |  |
|  | Fixed effects coefficients (95% CIs) | | | | | | | |
|  | Name | Estimate | SE | tStat | DF | P value | Lower | Upper |
|  | Intercept | 0.303 | 0.071 | 4.239 | 34 | 0.0001 | 0.157 | 0.448 |
|  | Environmental condition Impoverished | -0.071 | 0.055 | -1.303 | 34 | 0.201 | -0.184 | 0.04 |
|  | Trial number (1 or 2) | 0.137 | 0.038 | 3.59 | 34 | 0.001 | 0.059 | 0.215 |
| Response | AIC | BIC | LogLikelihood | Deviance |  |  |  |  |
| Exploration (N=40) | -8.551 | 3.295 | 9.275 | -18.551 |  |  |  |  |
|  | Fixed effects coefficients (95% CIs) | | | | | | | |
|  | Name | Estimate | SE | tStat | DF | P value | Lower | Upper |
|  | Intercept | 0.416 | 0.071 | 5.854 | 76 | 1.145e-07 | 0.274 | 0.558 |
|  | Environmental condition | -0.014 | 0.074 | -0.199 | 76 | 0.842 | -0.163 | 0.133 |
|  | Trial number (1 or 2) | 0.160 | 0.031 | 5.111 | 76 | 2.312e-06 | 0.097 | 0.222 |
| Response | AIC | BIC | LogLikelihood | Deviance |  |  |  |  |
| Exploration (N=19) | 6.103 | 14.158 | 1.948 | -3.896 |  |  |  |  |
|  | Fixed effects coefficients (95% CIs) | | | | | | | |
|  | Name | Estimate | SE | tStat | DF | P value | Lower | Upper |
|  | Intercept | 0.392 | 0.113 | 3.444 | 34 | 0.001 | 0.161 | 0.623 |
|  | Environmental condition Impoverished | -0.003 | 0.103 | -0.033 | 34 | 0.973 | -0.213 | 0.206 |
|  | Trial number (1 or 2) | 0.190 | 0.055 | 3.463 | 34 | 0.001 | 0.078 | 0.302 |
| Response | AIC | BIC | LogLikelihood | Deviance |  |  |  |  |
| Activity level (N=40) | -874.680 | -862.840 | 442.360 | -884.680 |  |  |  |  |
|  | Fixed effects coefficients (95% CIs) | | | | | | | |
|  | Name | Estimate | SE | tStat | DF | P value | Lower | Upper |
|  | Intercept | 0.002 | 0.0002 | 8.993 | 76 | 1.371e-13 | 0.002 | 0.003 |
|  | Environmental condition | 0.000 | 0.0002 | 0.358 | 76 | 0.721 | -0.0004 | 0.0006 |
|  | Trial number (1 or 2) | -0.000 | 0.0001 | -2.175 | 76 | 0.032 | -0.0006 | -2.78e-05 |
| Response | AIC | BIC | LogLikelihood | Deviance |  |  |  |  |
| Activity level (N=19) | -399.320 | -391.270 | 204.660 | -409.320 |  |  |  |  |
|  | Fixed effects coefficients (95% CIs) | | | | | | | |
|  | Name | Estimate | SE | tStat | DF | P value | Lower | Upper |
|  | Intercept | 0.002 | 0.0005 | 5.118 | 34 | 1.205e-05 | 0.001 | 0.003 |
|  | Environmental condition | 0.000 | 0.0003 | 1.186 | 34 | 0.243 | -0.0002 | 0.001 |
|  | Trial number (1 or 2) | -0.000 | 0.0002 | -0.419 | 34 | 0.677 | -0.0006 | 0.0004 |
