# Supplementary material for: Early experience affects foraging behavior of wild fruit bats more than their original behavioral predispositions
Source: eLife. 2025 Nov 11;14:RP103220. doi: 10.7554/eLife.103220 (PMC12604856; doi:10.7554/eLife.103220)
Supplement: Supplementary file 2. — DaysOutProportion ∼1 + EnvironmentalCondition + Sex + Age + (1∣Bat_ID) With Y ∼ Binomial (TotalExperimentDays) and link = logit. [file elife-103220-supp2.docx]

**Supplementary File 2.** GLMM results for proportion of nights foraging outside the roost (%).

DaysOutProportion ∼ 1 + EnvironmentalCondition + Sex + Age + (1∣Bat_ID)

With Y ∼ Binomial (TotalExperimentDays) and link=logit

| Response | AIC | BIC | LogLikelihood | Deviance |  |  |  |  |
| --- | --- | --- | --- | --- | --- | --- | --- | --- |
| Proportion of Nights Outside | -207.95 | -185.13 | 108.97 | -217.95 |  |  |  |  |
|  | Fixed effects coefficients (95% CIs) | | | | | | | |
|  | Name | Estimate | SE | tStat | DF | P value | Lower | Upper |
|  | Intercept | 1.315 | 0.419 | 3.134 | 705 | 0.001 | 0.491 | 2.139 |
|  | Environmental condition_Impoverished | 0.024 | 0.399 | 0.061 | 705 | 0.951 | -0.760 | 0.808 |
|  | Sex_M | 0.323 | 0.393 | 0.820 | 705 | 0.412 | -0.450 | 1.096 |
|  | Age | 6.77e-05 | 0.0003 | 0.179 | 705 | 0.857 | -0.0006 | 0.0008 |
